# The complete mitochondrial genome of the Arctic fairy shrimp *Branchinectapaludosa* (Müller, 1788) (Anostraca, Branchinectidae) from Sirius Passet, North Greenland

**DOI:** 10.3897/BDJ.10.e90200

**Published:** 2022-10-28

**Authors:** Ji-Hoon Kihm, Euna Jo, Tae-Yoon S Park, Bo-Mi Kim

**Affiliations:** 1 Division of Earth Sciences, Korea Polar Research Institute, Incheon, Korea, South Division of Earth Sciences, Korea Polar Research Institute Incheon Korea, South; 2 Division of Life Sciences, Korea Polar Research Institute, Incheon, Korea, South Division of Life Sciences, Korea Polar Research Institute Incheon Korea, South; 3 Division of Biotechnology, College of Life Sciences and Biotechnology, Korea University, Seoul, Korea, South Division of Biotechnology, College of Life Sciences and Biotechnology, Korea University Seoul Korea, South; 4 Polar Science, University of Science & Technology, Daejeon, Korea, South Polar Science, University of Science & Technology Daejeon Korea, South; 5 Research Unit of Cryogenic Novel Material, Korea Polar Research Institute, Incheon, Korea, South Research Unit of Cryogenic Novel Material, Korea Polar Research Institute Incheon Korea, South

**Keywords:** *Branchinectapaludosa*, Greenland anostraca, Branchinectidae, mitogenome, phylogeny

## Abstract

Here we report the complete mitochondrial genome of the Arctic fairy shrimp, *Branchinectapaludosa* (Müller, 1788) (Anostraca, Branchinectidae), which was collected in the High Arctic of North Greenland. A complete 16,059 bp mitochondrion of *B.paludosa* was sequenced and assembled with the Illumina next generation sequencing platform. The *B.paludosa* mitogenome contains 13 PCGs, 22 tRNAs and 2 rRNA genes that are commonly observed in most metazoans and shows the conserved gene arrangement pattern of Anostraca. Our results of the phylogenomic analysis are consistent with the previous phylogenetic relationship, based on nuclear 18S ribosomal DNA. The *B.paludosa* mitogenome will be useful for understanding the geographical distribution and phylogenetic relationship of anostracans.

## Introduction

Many branchiopod crustaceans inhabit harsh, hazardous and anomalous aquatic environments, which are even subject to drought and freeze episodes and, thus, they have frequently developed dormancy mechanisms for survival and population maintenance (Brendonck 1996). The family Branchinectidae was originally known as monogeneric with the genus *Branchinecta* (Rogers 2006), but a new genus *Archaebranchinecta* was subsequently recognised by its different gonopod and genital segment (Rogers and Coronel 2011). The genus *Branchinecta* consists of approximately 50 species, distributed in all continents, except for Africa and Australia (Rogers 2006). Despite their wide distribution, no complete mitochondrial genome sequence is available to date. The Arctic fairy shrimp, *Branchinectapaludosa* (Müller, 1788) is known as a circumpolar species inhabiting in Arctic ponds and lakes (Belk and Brtek 1995). The diapause eggs of this species normally hatch with snowmelt and fully develop during the short Arctic summer (Lindholm et al. 2015), being known as a cold stenothermic species (Lindholm et al. 2012). We here report the mitochondrial genome of *B.paludosa* collected from the High Arctic of North Greenland. This will not only be the first complete mitochondrial genome of the species that could be used for investigating the phylogenetic relationship with other anostracans and branchiopods, but also be the first genetic information from the High Arctic; previously, the sample from the northernmost habitat was collected from the latitude 75°N (Lindholm et al. 2016).

## Material and methods

An individual male specimen of *Branchinecta* was sampled from a small lake near Sirius Passet, North Greenland (82°47'7.7"N, 42°13'34.34"W) on 16 July 2017. With several morphological characters, this specimen was identified as *B.paludosa* (Müller, 1788): i.e. a longer proximal antennomere than a distal antennomere on the second antenna, spinose second antenna medial surface and a straight and triangular distal second antennal antennomere (Rogers and Aguilar 2020). This is the northernmost record of genus *Branchinecta* ever reported. The voucher specimen was registered in the Korea Polar Research Institute (KOPRI; Species ID:Anostraca1; Specimen ID:Anostraca1-2) and sequenced data were deposited in the Korea Polar Data Center (https://kpdc.kopri.re.kr/search/ad5267f7-ed99-4020-9b7a-1cd8880afe6a; Dr. Bo-Mi Kim; bomikim@kopri.re.kr). Total genomic DNA was extracted from a whole body of *B.paludosa* using the classical phenol/chloroform method (Kim et al. 2021). A genomic DNA library was prepared using TruSeq Nano DNA kit (Macrogen, Seoul, South Korea) according to the manufacturer's instructions (Illumina, San Diego, CA, USA). After removing adapter sequences, a total of 25,345,240 reads was produced by the Illumina HiSeq platform and *de novo* assembly was performed using SPAdes v.3.11.1 (Bankevich et al. 2012). Genomic features and annotations were predicted using MITOS2 (Bernt et al. 2013) and tRNAscan-SE 2.0 (Lowe and Eddy 1997). The gene annotation was further confirmed using NCBI-BLAST (http://blast.ncbi.nlm.nih.gov). Nucleotide sequences of the concatenated 13 protein-coding genes and 2 ribosomal RNAs of *B.paludosa* and those of 14 branchiopod species were used for phylogenetic analysis. A Maximum Likelihood tree was constructed using FastTree version 2.1.10 with default parameters (Price et al. 2009). All used mtgenome’s information is incorporated in Table 1.

## Results and Discussion

**Figure 1.**
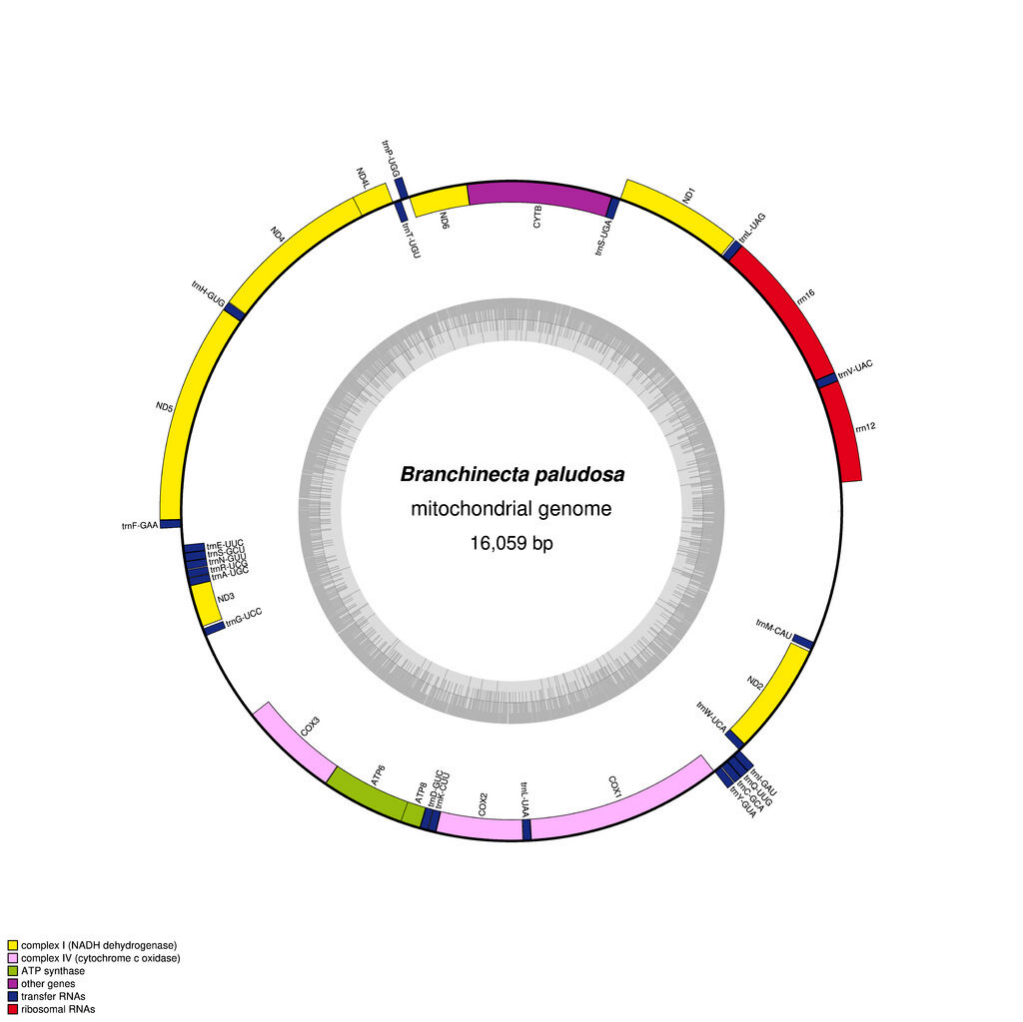
Circular map of the complete mitochondrial genome of *Branchinectapaludosa*. The map was drawn with OrganellarGenomeDRAW (OGDRAW) version 1.3.1 (Greiner et al. 2019).

**Figure 2.**
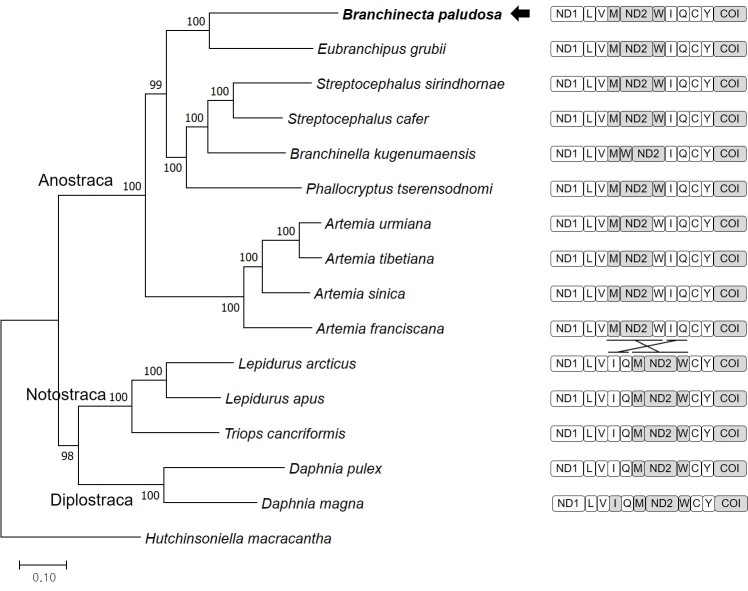
Maximum Likelihood phylogeny of 15 species of Branchiopoda with one species of Cephalocarida as an outgroup analysed with the concatenated nucleotide sequences of 13 PCGs and 2 rRNAs. Numbers on the branches indicate ML bootstrap percentages (100 replicates). The black arrow indicates the *B.paludosa* analysed in this study. A schematic diagram for the partial genomic structure of each mitogenome is appended on the right side of the phylogenetic tree. Other regions were omitted due to their same organisation. Genes on the major stand are shown in grey. Gene names for entire tRNAs are abbreviated as single-letter codes.

The assembly produced a complete consensus sequence with 16,059 bp, which contained 13 protein-coding genes (PCGs), 22 tRNAs, 2 rRNAs and one putative control region (MZ853171) (Fig. 1). Eleven PCGs (*ND1*, *ND6*, *ND4L*, *ATP8* and *COI* with ATT; *CYTB*, *ND4*, *ND3*, *COIII* and *COII* with ATG; *ND2* with ATC) have typical ATN as a start codon. However, two genes, *ND5* and *ATP6*, possess TTG and GTG as a start codon, respectively. Ten PCGs have TAA or TAG as a stop codon, whereas three genes, *CYTB*, *COII* and *COI* have an incomplete stop codon T (two nucleotides are missing). Two distinct gene arrangements in tRNA structures have been recognised in Crustacea: the ancestral pancrustacean pattern and the anostracan pattern (Cook et al. 2005) (Fig. 2). The gene arrangement of the *B.paludosa* tRNAs also complies with the typical pattern of anostracans as observed in the mitogenomes of *Eubranchipusgrubii*, two species of *Streptocephalus* and *Phallocryptustserensodnomi*. In anostracans mitogenomes, a gene coding for tRNA^Trp^ shows a rearrangement pattern in *Branchinellakugenumaensis*. Overall, this result supports the hypothesis of the ancestral gene rearrangement in anostracan mitochondrial genomes (Cook et al. 2005, Yang and Chen 2020).

The results of the phylogenetic analysis using mitogenome show that the *B.paludosa* is clustered within other anostracans, with *Eubranchipusgrubii* being the closest species (Fig. 2). The complete mitogenome of *B.paludosa* here in this study will provide essential information for understanding the potential correlation between the geographic distribution including the High Arctic and the phylogenetic relationship within Anostraca.

## Figures and Tables

**Table 1.** Taxonomy, mitogenome sizes, length of the input sequence for phylogetic analysis and GenBank accession numbers used in this study.

Order	Species	Mitogenome size (bp)	Length of PCGs+rRNAs (bp)	GenBank ID	Reference
Anostraca	*Artemiafranciscana*	15,822	12,452	NC_001620.1	Perez et al. 1994
Anostraca	*Artemiasinica*	15,689	12,397	NC_042147.1	Asem et al. 2019
Anostraca	*Artemiatibetiana*	15,742	12,439	NC_021383.1	Zhang et al. 2013
Anostraca	*Artemiaurmiana*	15,945	12,441	NC_021382.1	Zhang et al. 2013
Anostraca	*Branchinectapaludosa*	16,059	12,661	MZ853171	This study
Anostraca	*Branchinellakugenumaensis*	15,127	12,502	MN660045.1	Yang and Chen 2020
Anostraca	*Eubranchipusgrubii*	16,328	12,595	NC_050310.1	NC_050310.1
Anostraca	*Phallocryptustserensodnomi*	16,493	12,513	NC_026710.1	Fan et al. 2016
Anostraca	*Streptocephaluscafer*	17,020	12,574	NC_046688.1	Tladi et al. 2020
Anostraca	*Streptocephalussirindhornae*	16,887	12,634	NC_026704.1	Liu et al. 2016
Brachypoda	*Hutchinsoniellamacracantha*	16,491	13,329	AY456189.1	Lavrov et al. 2004
Diplostraca	*Daphniamagna*	14,948	13,210	NC_026914.1	Cheng et al. 2016
Diplostraca	*Daphniapulex*	15,333	13,143	NC_000844.1	Crease 1999
Notostraca	*Lepidurusapus*	15,635	13,175	NC_044646.1	Luchetti et al. 2019
Notostraca	*Lepidurusarcticus*	15,223	13,181	NC_044654.1	Luchetti et al. 2019
Notostraca	*Triopscancriformis*	15,101	13,167	NC_004465.1	Luchetti et al. 2019
